# Simvastatin Downregulates Cofilin and Stathmin to Inhibit Skeletal Muscle Cells Migration

**DOI:** 10.3390/ijms23052848

**Published:** 2022-03-05

**Authors:** Li-Ping Lin, Tung-Yang Yu, Hsiang-Ning Chang, Wen-Chung Tsai, Jong-Hwei S. Pang

**Affiliations:** 1 Graduate Institute of Clinical Medical Sciences, Chang Gung University, Taoyuan City 33302, Taiwan; d0500103@cgu.edu.tw; 2 Department of Physical Medicine and Rehabilitation, Chang Gung Memorial Hospital, Taoyuan City 33302, Taiwan; mr3964@cgmh.org.tw (T.-Y.Y.); changhn@cgmh.org.tw (H.-N.C.); 3 School of Medicine, Chang Gung University, Taoyuan City 33302, Taiwan

**Keywords:** simvastatin, skeletal muscle cells, cell migration, cell proliferation, cofilin, stathmin

## Abstract

Statins are the most effective therapeutic agents for reducing cholesterol synthesis. Given their widespread use, many adverse effects from statins have been reported; of these, musculoskeletal complications occurred in 15% of patients after receiving statins for 6 months, and simvastatin was the most commonly administered statin among these cases. This study investigated the negative effects of simvastatin on skeletal muscle cells. We performed RNA sequencing analysis to determine gene expression in simvastatin-treated cells. Cell proliferation and migration were examined through cell cycle analysis and the transwell filter migration assay, respectively. Cytoskeleton rearrangement was examined through F-actin and tubulin staining. Western blot analysis was performed to determine the expression of cell cycle-regulated and cytoskeleton-related proteins. Transfection of small interfering RNAs (siRNAs) was performed to validate the role of cofilin and stathmin in the simvastatin-mediated inhibition of cell migration. The results revealed that simvastatin inhibited the proliferation and migration of skeletal muscle cells and affected the rearrangement of F-actin and tubulin. Simvastatin reduced the expression of cofilin and stathmin. The knockdown of both cofilin and stathmin by specific siRNA synergistically impaired cell migration. In conclusion, our results indicated that simvastatin inhibited skeletal muscle cell migration by reducing the expressions of cofilin and stathmin.

## 1. Introduction

Statins are inhibitors of 3-hydroxy-3-methylglutaryl coenzyme A (HMG-CoA) reductase and the most effective therapeutic agents used to reduce cholesterol synthesis and the low-density lipoprotein level in blood [1,2,3]. Statins are used for the treatment and prevention of hyperlipidemia and can reduce the incidence of cardiovascular events in patients. However, as the use of statins has become widespread, many adverse effects have been reported, especially statin-associated myopathy, such as muscle cramping, soreness, fatigue, weakness, myositis, and rhabdomyolysis. A study reported that 10–15% of patients developed musculoskeletal complications after receiving statins for 6 months [4]. The side effects of statins often become apparent during or after strenuous bouts of exercise [5,6]. Among statin-associated myopathy, muscle weakness was observed in 58% of patients receiving statins, and simvastatin was the most commonly administered statin among these patients (49% of patients) [7]. The FDA Adverse Event Reporting System database reported that 36% of statin-associated rhabdomyolysis was associated with simvastatin from November 1997 through March 2000 [8].

It has been reported that statins impair skeletal muscle healing on postinjury [9,10]. Muscle healing is a complex process that involves inflammatory, repair, and remodeling phases. In the inflammatory phase, neutrophils and macrophages invade the injured site from 12 h to 4 days after injury. These inflammatory cells remove necrotic muscle cells through phagocytosis and release growth factors and cytokines, including insulin-like growth factor 1, transforming growth factor-β, interferon-γ, and tumor necrosis factor. These factors affect the proliferation of myogenic precursor cells and, thus, their population [11]. Myogenic precursor cells (also known as satellite cells) are located between the basal lamina and myofibers. In the repair phase, satellite cells begin to proliferate, migrate to injured sites, and differentiate into multinucleated myotubes and, eventually, into myofibers [12]. 

Cell proliferation plays a key role in the healing process of injured muscle. Cell proliferation is governed by the eukaryotic cell cycle [13], which is regulated by various signals that inhibit cell cycle progression. Similar to other types of cells, the cell division cycle of skeletal muscle cells has four stages: gap 1 (G1), synthesis (S), gap 2 (G2), and mitosis (M). This tightly controlled temporal order is controlled by the sequential activation of various protein kinases known as cyclin-dependent kinases (CDKs) that form a complex with various cyclins [14]. Cell migration is a crucial factor for muscle healing and a complicated cellular behavior driven by the cytoskeleton. The cytoskeleton, which includes actin microfilaments, microtubules, and intermediate filaments, plays a crucial role in the cell migration process. Cell migration can be divided into three stages: protrusion (spreading), attachment, and traction. In the protrusion phase, the lamellipodia and filopodia are extended forward over the extracellular matrix [15,16]. The lamellipodia and filopodia are driven by the dynamic transition of actin microfilaments (monomeric actin [G-actin] and numerous actin filaments [F-actin]); F-actin bundles mediate the initiation and orientation of the lamellipodia [17]. The dynamic regulation of G-actin and F-actin is driven by cofilin, which is a member of the actin depolymerizing factor/cofilin family [18]. Microtubules are composed of α and β tubulins, which are organized in a polarized manner, and can regulate protrusion and focal adhesion in the migration process [19]. Stathmin is a key regulator of microtubule polymerization and depolymerization in the migration process and reorganizes the mitotic spindle by destabilizing microtubules [20,21].

In the present study, we hypothesized that simvastatin impairs skeletal muscle healing by downregulating cytoskeleton-associated proteins. This study investigated the effects of simvastatin on the migratory ability of skeletal muscle cells and the underlying molecular mechanism.

## 2. Results

### 2.1. Simvastatin Moderated the Expression of Genes Related to Cell Proliferation and Migration in Skeletal Muscle Cells

**Figure 1:**
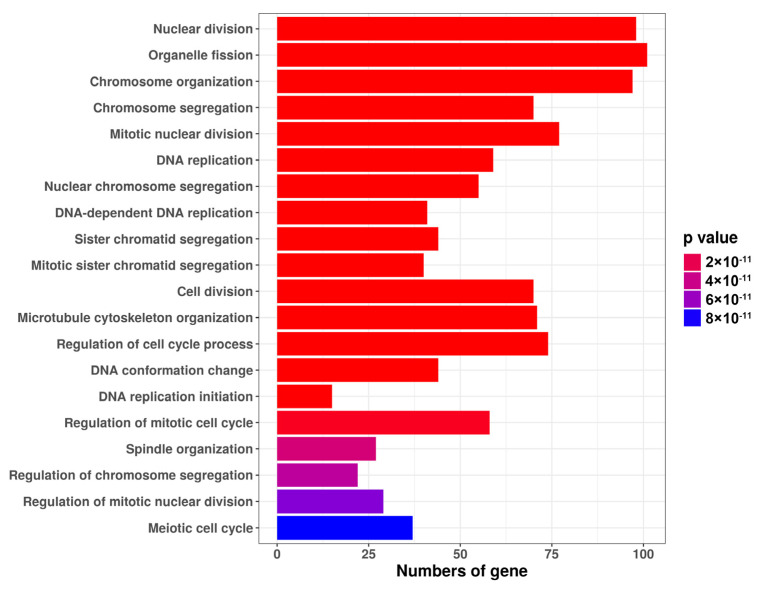
Simvastatin inhibited the expression of genes related to cell proliferation and migration in skeletal muscle cells. Gene ontology enrichment analysis of DEGs downregulated by simvastatin in skeletal muscle cells. The color scale indicated the *p* values; the x-axis indicated the DEGs numbers in the biological process section of the gene ontology database.

To investigate the effect of simvastatin on skeletal muscle cells, we performed RNAsequencing to analyze gene expression in skeletal muscle cells treated with 10 μM simvastatin. A total of 2355 differentially expressed genes (DEGs) were identified between control group and 10 μM simvastatin group. Simvastatin upregulated and downregulated the expression of 968 and 1387 genes, respectively, compared with control (Supplementary Figure S1). The results of Gene ontology (GO)enrichment analysis revealed that DEGs downregulated by simvastatin were related to nuclear division, DNA replication, and microtubule cytoskeleton organization (Figure 1). The results indicated that simvastatin inhibited the expression of genes related to cell proliferation and migration.

### 2.2. Simvastatin Affected Cell Proliferation by Downregulating Cyclin E, Cyclin A, and Cdk1

**Figure 2:**
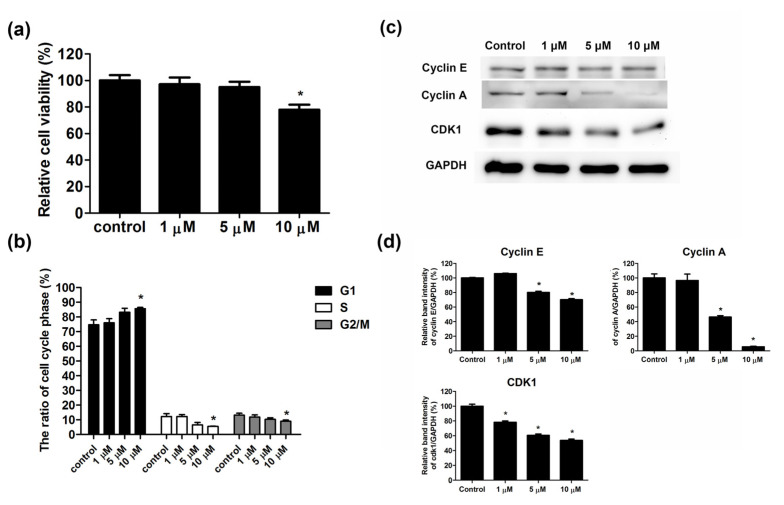
Simvastatin inhibited the proliferation of skeletal muscle cells. Skeletal muscle cells were untreated or treated with 1, 5, or 10 μM simvastatin for 24 h. (**a**) Cell viability was determined using the MTT assay. (**b**) Cell cycle analysis was performed. (**c**) Cell extracts were examined through Western blot analysis. (**d**) The band intensities of cyclin E, cyclin A, and CDK1are shown. GAPDH was used as the internal control. Data are presented as the mean ± standard error of the mean of six independent experiments. * *p* < 0.05.

The findings of RNAsequencing analysis indicated that simvastatin inhibited the expression of cell cycle regulation genes, namely cyclin E, cyclin A, and Cdk1. Furthermore, we investigated the effect of simvastatin on cell viability and the cell cycle. Skeletal muscle cells were treated with varying concentrations of simvastatin for 24 h; the MTT assay was performed to examine the viability of skeletal muscle cells. The results revealed that the viability of skeletal muscle cells decreased after simvastatin treatment. The relative cell viability was 100.0% ± 4.0% in control cells and 97.2% ± 5.1%, 95.1% ± 3.9%, and 78.0% ± 3.7% in cells treated with 1, 5, and 10 μM simvastatin, respectively (Figure 2a). The results of cell cycle analysis indicated that simvastatin caused G1 phase arrest and inhibited the S and G2 phases of skeletal muscle cells (Figure 2b). We observed that 74.6% ± 3.3% of control cells were in the G1 phase and 76.0% ± 2.8%, 83.2% ± 2.7%, and 85.5% ± 1.0% of skeletal muscle cells treated with 1, 5, and 10 μM simvastatin were in the G1 phase, respectively. Furthermore, 12.3% ± 2.0% of control cells were in the S phase, and 12.2% ± 1.3%, 6.5% ± 1.7%, and 5.5% ± 0.2% of skeletal muscle cells treated with 1, 5, and 10 μM simvastatin were in the S phase, respectively. We noted that 13.1% ± 1.4% of control cells were in the G2 phase and 11.8% ± 1.5%, 10.3% ± 1.0%, and 9.0% ± 0.8% of skeletal muscle cells treated with 1, 5, and 10 μM simvastatin were in the G1 phase, respectively. The protein expression of cyclin E, cyclin A, and CDK1 was determined through Western blot analysis (Figure 2c); the band intensity is shown in Figure 2d. The results revealed that simvastatin suppressed the protein expression of cylin E, cyclin A, and CDK1.

### 2.3. Simvastatin Reduced the Migration Ability of Skeletal Muscle Cells

**Figure 3:**
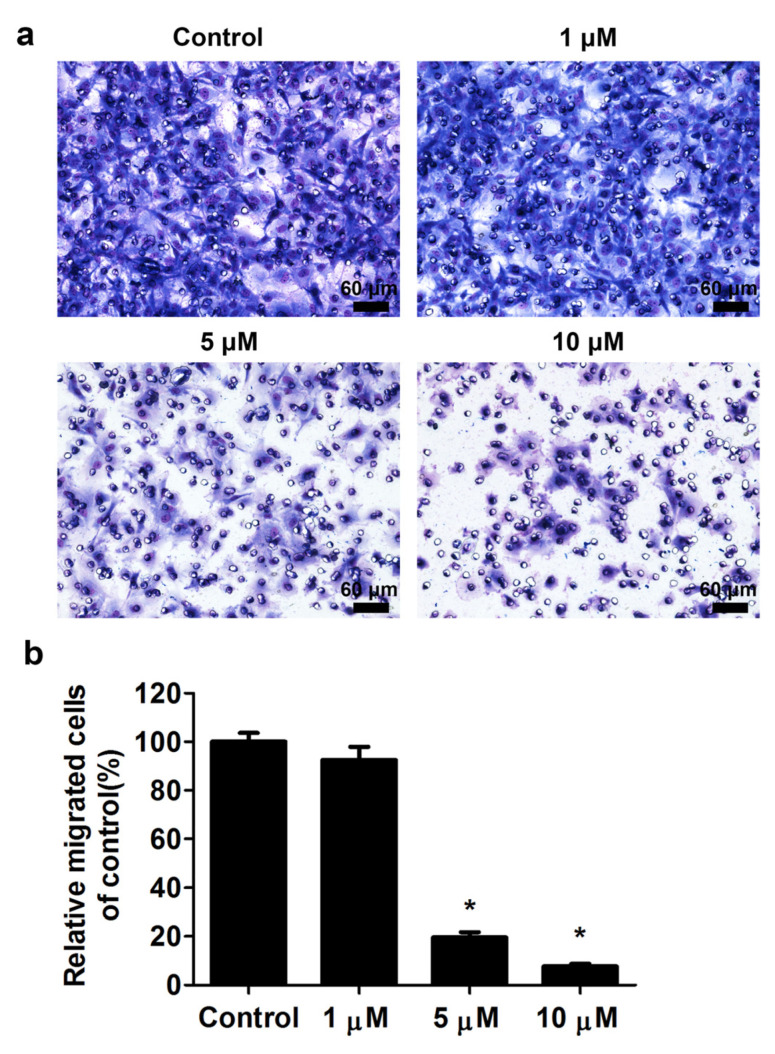
Simvastatin inhibited the migration ability of skeletal muscle cells. (**a**) Skeletal muscle cells were untreated or treated with 1, 5, or 10 μM simvastatin for 24 h. Cell migration ability was determined using the transwell filter migration assay. (**b**) Results are presented as the mean ± standard error of the mean of six independent experiments. * mean *p* < 0.05 compared with control. Scale bars: 60 μm.

As shown in Figure 2, simavastatin inhibited the proliferation of skeletal muscle cells. We next performed the transwell filter migration assay to evaluate the migration ability of skeletal muscle cells treated with simvastatin for 24 h. The results revealed that simvastatin reduced the migration ability of skeletal muscle cells (Figure 3a). The relative migration rate was 100.0% ± 3.6% in control cells and 92.4% ± 5.5%, 19.6% ± 2.2%, and 7.7% ± 1.1% in cells treated with 1, 5, and 10 μM simvastatin (Figure 3b). The differences between the groups were statistically significant (*p* < 0.05).

### 2.4. Simvastatin Inhibited the Spreading of Skeletal Muscle Cells

**Figure 4:**
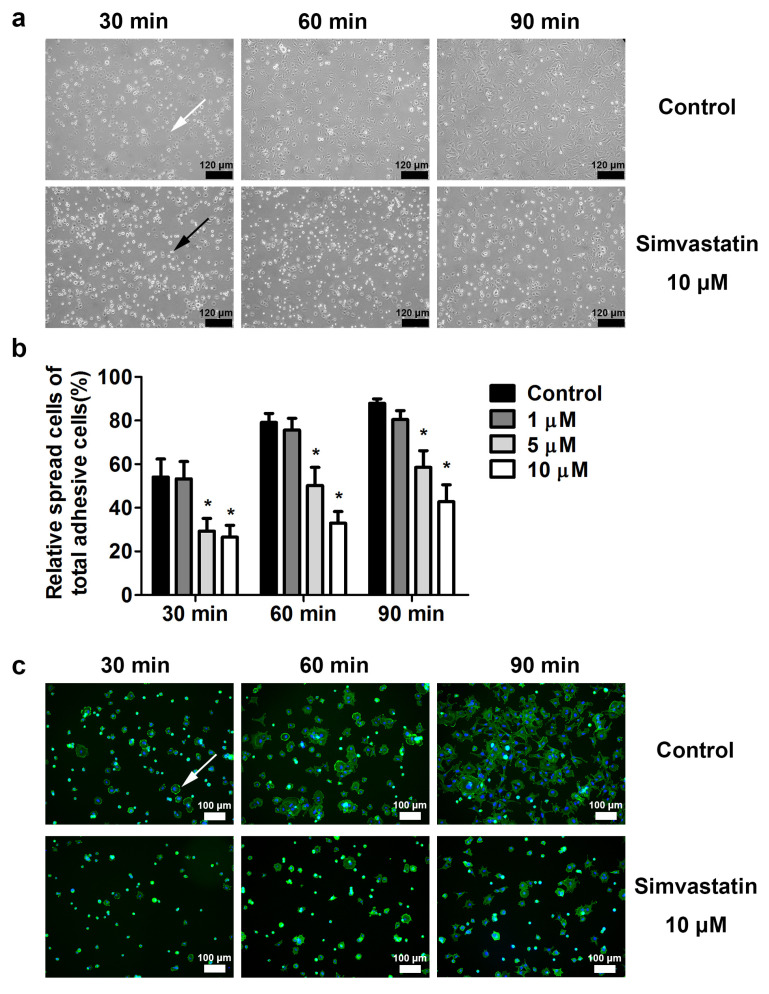
Simvastatin inhibited the spreading of skeletal muscle cells. (**a**) Skeletal muscle cells were untreated or treated with 1, 5, or 10 μM simvastatin for 24 h and plated on culture dishes. After plating for 30, 60, and 90 min, the attached cells started to spread out and were photographed at 100×. The numbers of spreading cells were decreased after simvastatin treatment. The spreading and nonspreading cells are indicated by white and black arrows, respectively. Scale bars: 120 μm. (**b**) The ratio of spreading cells to adhesion cells was decreased after simvastatin treatment. Data are presented as the mean ± standard error of the mean of six independent experiments. * mean *p* < 0.05 compared with control. (**c**) F-actin was stained green; nuclei were stained blue. Scale bars: 100 μm. The spread cells are indicated by white arrows.

As shown in Figure 3, simvastatin reduced the migration ability of skeletal muscle cells. The initial step of cell migration is cell attachment and spreading. We examined the cell spreading of skeletal muscle cells after simvastatin treatment (Figure 4a). The numbers of spreading and adhesive cells were separately counted at 30, 60, and 90 min after plating. At 90 min, the relative percentage of spreading was 87.9% ± 2.1% for control cells and 80.4% ± 4.2%, 58.5% ± 7.7%, and 42.8% ± 7.8% for cells treated with 1, 5, and 10 μM simvastatin, respectively (*p* < 0.05; Figure 4b). In addition, the F-actin distribution range of spreading cells was wider than that of nonspreading cells. The results indicated that simvastatin reduced cell spreading characterized by the appearance of pseudopodia extensions (Figure 4c).

### 2.5. Effect of Simvastatin on the Cytoskeleton Distribution of Skeletal Muscle Cells

**Figure 5:**
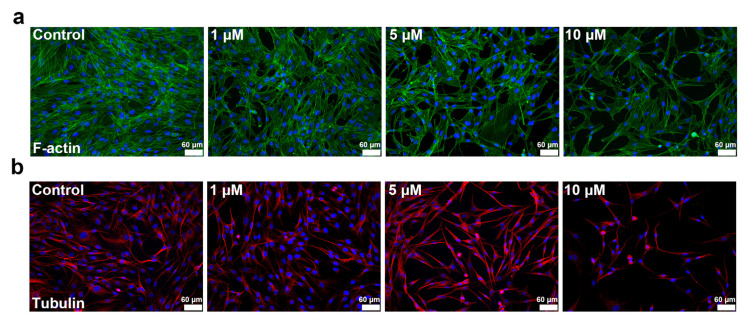
Simvastatin affected the distribution of F-actin and tubulin. Skeletal muscle cells were untreated or treated with 1, 5, or 10 μM simvastatin for 24 h. (**a**) The formation of F-actin was decreased after simvastatin treatment. F-actin was stained green, and nuclei were stained blue. (**b**) Tubulin was stained red, and nuclei were stained blue. The photographs were obtained using a fluorescence microscope (200×). Scale bars: 60 μm.

During the cell migration process, migrating cells are driven by cytoskeleton-associated proteins, namely F-actin and tubulin. We performed immunofluorescence staining to observe F-actin and tubulin in skeletal muscle cells treated with simvastatin. The distributions of F-actin and tubulin in skeletal muscle cells both changed after simvastatin treatment. The expression of F-actin was decreased after simvastatin treatment and was significantly decreased in cells treated with 10 μM simvastatin (Figure 5a). However, the formation of tubulin exhibited differences after simvastatin treatment. Tubulin became longer and bundled in the high-dose group (5 and 10 μM; Figure 5b). The results indicated that simvastatin changed the organization of F-actin and tubulin.

### 2.6. Simvastatin Reduced the Expression of Cytoskeleton-Associated Proteins in Skeletal Muscle Cells

**Figure 6:**
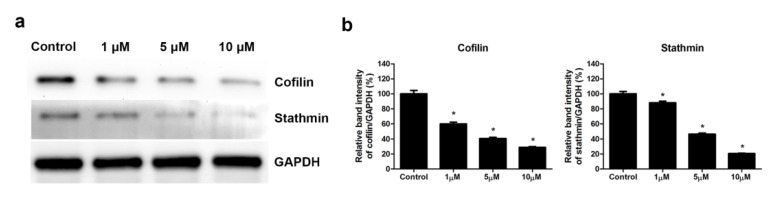
Simvastatin inhibited the expression of cytoskeleton-associated proteins in skeletal muscle cells. (**a**) Skeletal muscle cells were untreated or treated with 1, 5, or 10 μM simvastatin for 24 h, and cell extracts were analyzed through Western blot analysis. (**b**) The band intensities of cofilin and stathmin are shown. GAPDH was used as the internal control. Data are presented as the mean ± standard error of the mean of three independent experiments. * mean *p* < 0.05 compared with control.

Skeletal muscle cells were untreated or treated with 1, 5, or 10 μM simvastatin; the protein extracts were analyzed through Western blot analysis. The protein expressions of stathmin and cofilin are shown in Figure 6a. The band intensity of cofilin was 100.0% ± 4.6% in control cells and 60.0% ± 2.3%, 40.6% ± 1.6%, and 28.8% ± 1.1% in cells treated with 1, 5, and 10 μM simvastatin, respectively. The band intensity of stathmin was 100.0% ± 3.2% in control cells and 88.2% ± 2.1%, 46.4% ± 1.5%, and 20.5% ± 0.6% in cells treated with 1, 5, and 10 μM simvastatin, respectively (Figure 6b). The results of Western blot and band intensity analyses indicated that simvastatin suppressed the protein expression of cofilin and stathmin. 

### 2.7. Simvastatin-Mediated Inhibition of Cell Migration was Regulated by Cofilin and Stathmin

**Figure 7:**
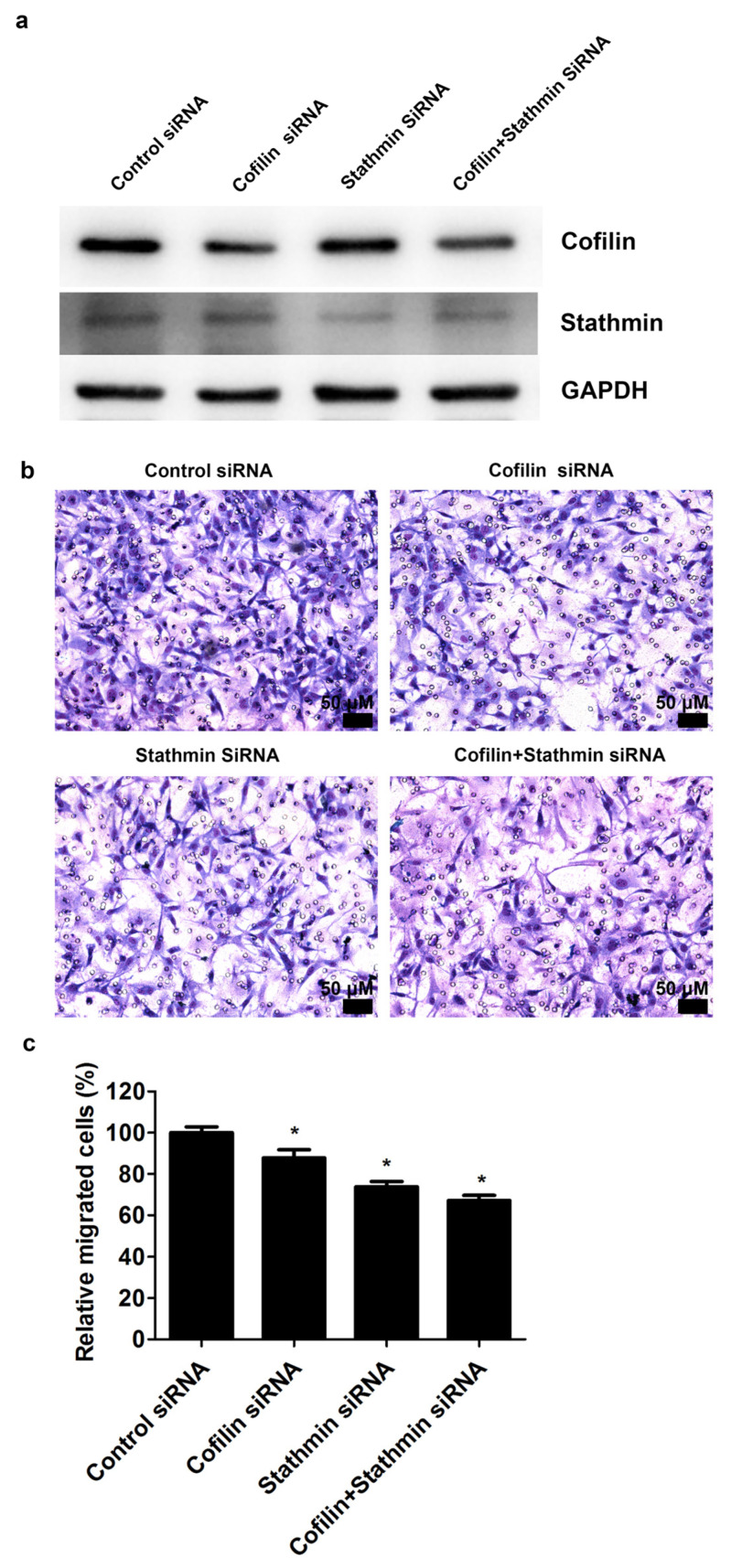
Knockdown of cofilin and stathmin inhibited the migration ability of skeletal muscle cells. Skeletal muscle cells were transfected with cofilin siRNA, stathmin siRNA, cofilin plus stathmin siRNA, or control siRNA. (**a**) The expression of cofilin and stathmin was analyzed through Western blot analysis. (**b**) The cell migration ability was determined using the transwell filter migration assay. (**c**) Data are presented as the mean ± standard error of the mean of four independent experiments. * mean *p* < 0.05 compared with control. Scale bars: 50 μm.

To examine the roles of cofilin and stathmin in cell migration, cofilin and stathmin were knocked down using specific siRNAs. The protein expression of cofilin and stathmin was decreased in the cofilin and stathmin siRNA groups, respectively, and downregulated in the cofilin plus stathmin siRNA group (Figure 7a). Furthermore, the migration ability of skeletal muscle cells transfected with cofilin siRNA, stathmin siRNA, and cofilin siRNA plus stathmin siRNA was significantly decreased (Figure 7b). The relative cell migration rates were 100.0% ± 2.9%, 87.7% ± 4.0%, 73.7% ± 2.6%, and 67.1% ± 2.5% in the control siRNA group, cofilin siRNA group, stathmin siRNA group, and cofilin plus stathmin siRNA group, respectively (*p* < 0.05, Figure 7c). 

## 3. Discussion

The potential mechanisms of statin-induced myopathy include reduced levels of isoprenoids, mitochondria dysfunction, and genetic factor. The isoprenoids are intermediates of the mevalonate pathway. Statin inhibited HMG-CoA reductase in cholesterol biosynthesis and reduced isoprenoid compounds. The isoprenoids, such as geranylgeranyl pyrophosphate (GGPP) and farnesyl pyrophosphate, dolichols and ubiquinone (coenzyme Q10), are involved in posttranslational modifications of small GTPases, protein glycosylation, mitochondria biogenesis, which is essential for cell growth, gene expression, and cytoskeleton assembly [4,22]. Most of the small GTPases, such as the Rho and Ras families, are related to cell behaviors, including cell proliferation, cell migration, and apoptosis. Several studies have reported that simvastatin inhibited the proliferation and migration of cancer cells [23,24,25,26]. The proliferation of skeletal muscle cells is regulated by the eukaryotic cell cycle [13]. Similar to other types of cells, muscle cells have four cell division cycle stages: G1, S, G2, and M. The chromosomal DNA of cells replicates during the S phase. When the cell cycle moves from the S to G2–M stages, cells begin to divide. The cell cycle is accurately controlled by cyclins and Cdk complexes [14]. Our previous study indicated that simvastatin suppressed tendon cell proliferation. Simvastatin reduced the expression of cyclins and cdks and altered cell cycle progression. We observed that the supplementation of GGPP reversed the inhibition of cell proliferation in tendon cells [27]. In this study, simvastatin reduced the viability of skeletal muscle cells. The GO enrichment analysis indicated that DEGs downregulated by simvastatin were related to nuclear division, chromosome organization, DNA replication, and regulation of mitotic cell cycle. Moreover, simvastatin inhibited skeletal muscle cell proliferation and caused cell cycle arrest at the G1/S transition. The underlying molecular mechanism appears to be related to the downregulation of cyclin E, cyclin A, and Cdk1. 

The GO enrichment analysis also revealed that DEGs downregulated by simvastatin were related to microtubule cytoskeleton organization. A Study indicated that simvastatin inhibits the migration and adhesion of monocytes and disorganizes F-actin in endothelial cells [28]. In this study, simvastatin reduced the migration and spreading of skeletal muscle cells. F-actin organization was inhibited during the spreading process (Figure 2). We performed immunofluorescence staining to examine the cytoskeleton organization after simvastatin treatment, including that of actin microfilaments (F-actin) and microtubules (tubulin). F-actin was decreased; tubulin clustered together after simvastatin treatment. The results indicated that simvastatin likely affected the cytoskeleton organization of skeletal muscle cells. The results of this study revealed that simvastatin downregulated the expression of cofilin and stathmin. Cofilin depolymerizes F-actin to G-actin by binding to the ADP-bound actin of F-actin. The activity of cofilin was regulated by the phosphorylation and dephosphorylation of serine 3 residues [29]. Several studies have indicated that the defect in cofilin inhibited cell spreading and migration in cancer metastasis [30,31]. Furthermore, a previous study indicated that simvastatin reduced the expression of F-actin and attenuated neuropathic pain by inhibiting the RhoA/LIMK/cofilin pathway in a chronic neuropathic pain model [32]. Stathmin is a key regulator that depolymerizes microtubules. Phosphorylation at Ser 16 and Ser 38 inactivates stathmin and stabilizes microtubules; the phosphorylation of stathmin is regulated by CDKs. Moreover, the signal transducer and activator of transcription was associated with the activity of stathmin as an antagonist in microtubule polymerization [33]. Stathmin was observed to be frequently overexpressed in breast cancer cells and to mediate the resistance of antimicrotubule agents that are used as chemotherapy drugs [34]. However, the knockdown of stathmin expression reduced cell migration in esophageal squamous cell carcinoma cells and neuroblastoma cells [35,36]. Stathmin is involved in keratinocyte migration during cutaneous regeneration [37]. We used specific siRNAs to knockdown the expression of cofilin and stathmin. The cell migration rates of the cofilin and stathmin siRNA groups were lower than that of the control siRNA group. However, the cell migration rate of the cofilin plus stathmin siRNA group was lower than that of the single siRNA group. The results indicated that the lack of cofilin and stathmin in skeletal muscle cells inhibited cell migration ability. In conclusion, simvastatin inhibited the proliferation of skeletal muscle cells by downregulation of cyclin E, cyclin A, and Cdk1, and inhibited migration of skeletal muscle cells by downregulating cofilin and stathmin. This might be a potential mechanism of statin-induced myopathy that exerts a negative effect on muscle healing.

The results of this study revealed that simvastatin downregulated the expression of cofilin and stathmin. Cofilin depolymerizes F-actin to G-actin by binding to the ADP-bound actin of F-actin. The activity of cofilin was regulated by the phosphorylation and dephosphorylation of serine 3 residues [27]. Several studies have indicated that the defect in cofilin inhibited cell spreading and migration in cancer metastasis [28,29]. Furthermore, a previous study indicated that simvastatin reduced the expression of F-actin and attenuated neuropathic pain by inhibiting the RhoA/LIMK/cofilin pathway in a chronic neuropathic pain model [30]. Stathmin is a key regulator that depolymerizes microtubules. Phosphorylation at Ser 16 and Ser 38 inactivates stathmin and stabilizes microtubules; the phosphorylation of stathmin is regulated by CDKs. Moreover, the signal transducer and activator of transcription was associated with the activity of stathmin as an antagonist in microtubule polymerization [31]. Stathmin was observed to be frequently overexpressed in breast cancer cells and to mediate the resistance of antimicrotubule agents that are used as chemotherapy drugs [32]. However, the knockdown of stathmin expression reduced cell migration in esophageal squamous cell carcinoma cells and neuroblastoma cells [33,34]. Stathmin is involved in keratinocyte migration during cutaneous regeneration [35]. We used specific siRNAs to knockdown the expression of cofilin and stathmin. The cell migration rates of the cofilin and stathmin siRNA groups were lower than that of the control siRNA group. However, the cell migration rate of the cofilin plus stathmin siRNA group was lower than that of the single siRNA group. The results indicated that the lack of cofilin and stathmin in skeletal muscle cells inhibited cell migration ability. In conclusion, simvastatin inhibited the spreading and migration of skeletal muscle cells by downregulating cofilin and stathmin and might exert a negative effect on muscle healing.

## 4. Materials and Methods

### 4.1. Animals

Sprague-Dawley (SD) rats (male, 250 g) were obtained from BioLASCO Taiwan Co., Ltd. Taipei, Taiwan. All animal experimental procedures were approved by the Institutional Animal Care and Use Committee of Chang Gung University (CGU16-026).

### 4.2. Primary Culture of Rat Skeletal Muscle Cells

Skeletal muscle cells were isolated from the gastrocnemius muscle of SD rats. The isolation method was described previously [38,39]. The gastrocnemius muscle was treated with 2 mg/mL of type I collagenase (Sigma-Aldrich, St. Louis, MO, USA) for 1 h at 37 °C in a humidified incubator (5% CO_2_/95% air), followed by treatment with 0.25% trypsin–EDTA for 1 h. The supernatant was filtered using a 70-μm cell strainer. After centrifugation (1500 rpm, 5 min), cell pellets were suspended in Dulbecco’s modified Eagle’s medium (DMEM) containing 10% fetal bovine serum (FBS) and 5% chick embryo extract (Gibco, Thermo Fisher Scientific, Waltham, MA, USA) and were seeded on a culture plate. After 1 h, the supernatant containing skeletal muscle cells was transferred to another culture plate and incubated at 37 °C in a humidified incubator (5% CO_2_/95% air). These cells were used in the subsequent experiments.

### 4.3. RNA Sequencing Analysis

Skeletal muscle cells were untreated or treated with 10 μM simvastatin (Sigma-Aldrich, St. Louis, MO, USA) for 24 h; total RNA was extracted using Trizol (Thermo Fisher Scientific, Waltham, MA, USA). RNA sequencing analysis was performed using Illumina NovaSeq 6000 (Illumina, Inc., San Diego, CA, USA). Differential expression analysis between the control group and the simvastatin-treated group was performed using DESeq2 (version 1.16.0., available at http://www.bioconductor.org/packages/release/bioc/html/DESeq2.html. Accessed dates: 27 October 2021). An adjusted *p* value of <0.05 and a |log2fold change| of >1 served as cutoff criteria for the selection of differentially expressed genes (DEGs). Gene ontology (GO) enrichment was performed using DEGs (adjusted *p* value ≤ 0.05).

### 4.4. Cell Viability Testing

Skeletal muscle cells were treated with 1, 5, or 10 μM simvastatin for 24 h. Subsequently, 50 μg/mL of 3-(4,5-dimethylthiazol-2-yl)-2,5-diphenyltetrazolium bromide) (MTT) reagent (Sigma-Aldrich, St. Louis, MO, USA) was added to the culture plate, and the plate was incubated at 37 °C for 1 h. Dimethyl sulfoxide (Sigma-Aldrich, St. Louis, MO, USA) was added to dissolve formazan crystals; aliquots were transferred to a 96-well plate. Absorbance was immediately read at 595 nm by using a multiwell spectrophotometer (Victor X3; Perkin Elmer Inc., Waltham, MA, USA).

### 4.5. Cell Cycle Analysis

Skeletal muscle cells were treated with 1, 5, or 10 μM simvastatin for 24 h, washed with phosphate-buffered saline (PBS) two times, and fixed with 1 mL of 70% ethanol in PBS for 1 h at −20 °C. After centrifugation at 3000 rpm for 5 min, the pellet was resuspended in PBS containing 0.5% Triton X-100 (Sigma-Aldrich, St. Louis, MO, USA) and 0.05% RNase A (Sigma-Aldrich, St. Louis, MO, USA) and incubated at 37 °C for 1 h. The cell suspension was centrifuged, washed, and resuspended in 1 mL of 50 μg/mL propidium iodide solution in PBS for 20 min. The cells were analyzed through flow cytometry (FACSCalibur; Becton Dickinson, San Francisco, CA, USA).

### 4.6. Transwell Filter Migration Assay

Skeletal muscle cells were treated with 1, 5, or 10 μM simvastatin for 24 h. The cells were seeded in the inner chamber of transwell filters (8.0-μm pores; Costar, Cambridge, MA, USA) at a density of 1 × 10^5^. The inner chamber was filled with 200 μL of serum-free DMEM, whereas the outer chamber was filled with 600 μL of DMEM containing 20% FBS. After 3 h, the cells were stained with Liu’s stain and washed twice in PBS. The cells present on the upper surface of the filter were removed using a cotton swab. The cells present on the lower surface were counted in eight random microscopic fields (200×).

### 4.7. Cells Spreading Assay and F-Actin Staining

Skeletal muscle cells treated with or without simvastatin for 24 h were subcultured and plated on six-well culture plates. After plating for 30, 60, and 90 min, the cells were observed and photographed under a light microscope (100×; CKX53; OLYMPUS, Tokyo, Japan). The cells with a round shape and bright appearance were considered to be nonspreading cells. The cells exhibiting pseudopodia extensions were considered to be spreading cells. The numbers of spreading and nonspreading cells were calculated in four random microscopic fields. For F-actin staining, the cells were fixed in 10% formalin for 15 min after plating for 30, 60 or 90 min. The cells were permeabilized using 0.1% Triton-X 100 in PBS for 5 min. After washing three times with PBS, the cells were incubated in blocking solution (3% BSA in PBS) at room temperature for 30 min and were incubated for 1 h with phalloidin-conjugated fluorescein isothiocyanate (FITC; Sigma, St. Louis, MO, USA) diluted in blocking solution. The cells were then washed in PBS and stained with PBS containing 1 μg/mL DAPI (Thermo Fisher Scientific, Waltham, MA, USA) for 5 min. Subsequently, the cells were washed in PBS and were examined using the ZOE Fluorescent Cell Imager (175×; Bio-Rad, Hercules, CA, USA).

### 4.8. Immunofluorescence Staining

Skeletal muscle cells were seeded on glass coverslips. The cells were untreated or treated with simvastatin for 24 h and fixed in 10% formalin for 15 min. After washing three times with PBS, glass coverslips were incubated in blocking solution (3% BSA in PBS) at room temperature for 30 min and incubated for 2 h with phalloidin-conjugated FITC (Sigma-Aldrich, St. Louis, MO, USA) or anti-tubulin antibody (1/200 dilution, Thermo Fisher Scientific, Waltham, MA, USA) or anti-stathmin antibody (Cell Signaling Technology, Danvers, MA, USA) diluted in blocking solution. The signal was detected using antirabbit IgG Alexa Fluor 488 or antimouse IgG Alexa Fluor 594 (Thermo Fisher Scientific, Waltham, MA, USA). After washing in PBS, the cells were stained with PBS containing 300 nM DAPI for 5 min. After washing in PBS, the cells were observed under a fluorescence microscope (200×; Eclipse Ni-U; Nikon, Tokyo, Japan).

### 4.9. Western Blot Analysis

Total protein from skeletal muscle cells was extracted using lysis buffer (20 mM HEPES, 1 mM EDTA, 1 mM EGTA, 20 mM NaF, 1 mM Na_3_VO_4_, 1 mM Na_2_P_2_O_7_, 1 mM DTT, 0.5 mM PMSF, 1 μg/mL leupeptin, 1% Triton X-100) and a protease inhibitor cocktail (TAAR-BBI2, BIOTOOLS Co., Ltd., Taipei, Taiwan). The protein concentration of the cell extracts was determined using the Bradford assay (Bio-Rad Laboratories, Richmond, CA, USA). Subsequently, 10 μg of total protein was resolved through 10% sodium dodecyl sulfate–polyacrylamide gel electrophoresis and was transferred onto a polyvinylidene membrane by using TG buffer (BIOTOOLS Co., Ltd., Taipei, Taiwan). The membrane was incubated in blocking solution (5% BSA in TBST) at room temperature for 1 h and then incubated for 2 h in blocking solution containing an appropriate dilution of primary antibodies, namely anti-GAPDH (1/1000 dilution, Proteintech, Rosemont, IL, USA), anti-cyclin E1 (1/1000 dilution, Abclonal, Woburn, MA, USA), anti-cyclin A2 (1/1000 dilution, Proteintech, Rosemont, IL, USA), anti-cdk1(1/1000 dilution, Abclonal, Woburn, MA, USA), anti-cofilin (1/1000 dilution, Cell Signaling Technology, Danver, MA, USA), and anti-stathmin (1/500 dilution, Cell Signaling Technology, Danver, MA, USA). After washing, the membranes were incubated in TBST containing horseradish peroxidase (HRP)-conjugated anti-mouse IgG (Leinco Technologies, Inc., St. Louis, MI, USA) or HRP-conjugated anti-rabbit IgG (Cell Signaling Technology, Danver, MA, USA) for 1 h. The membranes were washed three times in TBST and developed using the Luminata Crescendo Western HRP substrate (Merck Millipore, Darmstadt, Germany). The signal was quantified using the iBright FL1500 Imaging System (Thermo Fisher Scientific, Waltham, MA, USA).

### 4.10. siRNA and Transfection

Specific siRNAs targeting rat cofilin1 (5′-CUCUCUAUGACGCAACCUATT-3′ and 5′-UAGGUUGCGUCAUAGAGAGTT-3′) and stathmin1 (5′-CCUGACAAAUAUUCUAGAATT-3′ and 5′-UUCUAGAAUAUUUGUCAGGTT-3′) were provided by BIOTOOLS Co., Ltd., Taipei, Taiwan. The control siRNA (5′-UUCUCCGAACGUGUCACGUTT-3′ and 5′-ACGUGACACGUUCGGAGAATT-3′) was used as a negative control. Subsequently, the siRNAs were transfected into skeletal muscle cells by using Lipofectamine 2000 (Thermo Fisher Scientific, Waltham, MA, USA) according to the manufacturer’s instructions. After 24 h, the transfected cells were analyzed through Western blotting analysis or the transwell filter migration assay.

### 4.11. Statistical Analysis

All data were expressed as the mean ± standard error of the mean. Comparisons between groups were performed using the Kruskal–Wallis test. The Mann–Whitney U test was used to identify differences between the groups. A *p* value of <0.05 indicated statistical significance.

## Data Availability

Data used for this study is available on request from the corresponding authors.

## Supplementary Materials

The following supporting information can be downloaded at: https://www.mdpi.com/article/10.3390/ijms23052848/s1.
