# Supplementary material for: Simvastatin Downregulates Cofilin and Stathmin to Inhibit Skeletal Muscle Cells Migration
Source: Int J Mol Sci. 2022 Mar 5;23(5):2848. doi: 10.3390/ijms23052848 (PMC8911248; doi:10.3390/ijms23052848)
Supplement: Supplementary file 1 [file ijms-23-02848-s001.zip › ijms-1566743-supplementary.pdf]

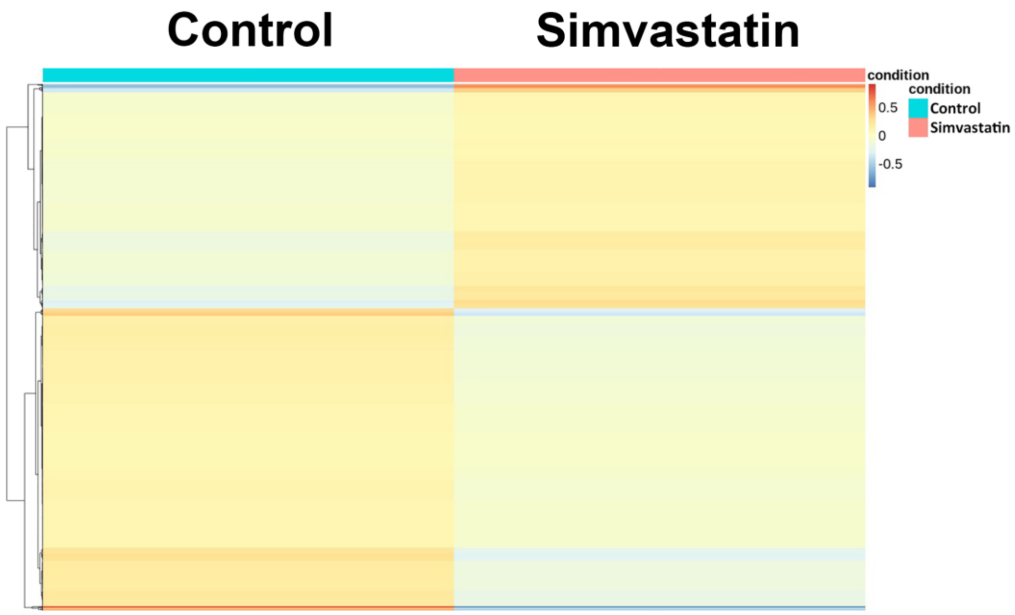

Figure S1. Heatmap of differentially expressed genes (DEGs) in simvastatin-treated skeletal muscle cells.
